# Editorial for the Special Issue “Nanoscale Ferroic Materials—Ferroelectric, Piezoelectric, Magnetic, and Multiferroic Materials”

**DOI:** 10.3390/nano12172951

**Published:** 2022-08-26

**Authors:** Changhong Yang, Chunchang Wang, Zhenxiang Cheng

**Affiliations:** 1 Shandong Provincial Key Laboratory of Preparation and Measurement of Building Materials, University of Jinan, Jinan 250022, China; 2 Laboratory of Dielectric Functional Materials, School of Materials Science & Engineering, Anhui University, Hefei 230601, China; 3 Institute for Superconducting and Electronic Materials, Australian Institute of Innovative Materials, Innovation Campus, University of Wollongong, Squires Way, North Wollongong, NSW 2500, Australia

Ferroic materials, including ferroelectric, piezoelectric, magnetic, and multiferroic materials, are receiving great scientific attentions due to their rich physical properties. They have shown their great advantages in diverse fields of application, such as information storage, sensor/actuator/transducers, energy harvesters/storage, and even environmental pollution control. At the same time, nanostructure has assumed an increasing importance as the key to the miniaturization of solid-state electronics, decrease of the power consumption, and reduction of production cost. At present, ferroic nanostructures have been widely acknowledged to advance and improve currently existing electronic devices, as well as to develop future ones.

This Special Issue, comprising ten research articles, covers the characterization of crystal and microstructure, the design and tailoring of ferro/piezo/dielectric, magnetic, and multiferroic properties, and the presentation of related applications. These papers present various kinds of nanomaterials, such as the ferroelectric/piezoelectric thin films, i.e., PMNPT, BiFeO_3_, BiOCl/NaNbO_3_, CH_3_NH_3_PbI_3_, Hf_0.5_Zr_0.5_O_2_, and CuInP_2_S_6_; dielectric storage thin film, i.e., Ba_0.5_Sr_0.5_TiO_3_/0.4BiFeO_3_-0.6SrTiO_3_, Bi(Fe_0.93_Mn_0.05_Ti_0.02_)O_3_-CaBi_4_Ti_4_O_15_ solid solution; dielectric gate layer, i.e., Sm_2_O_3_/Al_2_O_3_/InP; and magnonic metamaterials, i.e., ferromagnet/heavy metal bilayer. These nanomaterials are expected to have applications in ferroelectric non-volatile memory, ferroelectric tunneling junction memory, energy-storage pulsed-power capacitor, metal oxide semiconductor field-effect-transistor device, humidity sensor, environmental pollutant remediation, and spin-wave devices. The purpose of this Special Issue is to communicate the recent developments in the research of nanoscale ferroic materials. In the following part, we present a brief overview of the published articles and hope to provide a useful resource for potential readers.

Aiming at obtaining ferroelectric thin films with strong polarization feature, effective methods of chemical doping and regulating the preparation process are applied. Kim et al. fabricated Hf_0.5_Zr_0.5_O_2_ thin films via plasma-enhanced atomic layer deposition. A high remanent polarization with 2*P_r_* of 38.2 μC/cm^2^ and an excellent fatigue endurance of 2.5 × 10^7^ cycles were obtained by regulating the deposition temperature, post-annealing temperature and RF plasma discharge time [1]. Among the studies about Hf_x_Zr_1-x_O_2_, the work in this study showed relatively good remanent polarization and fatigue endurance performances despite being under the lowest deposition temperature. Feng et al. [2] focus their work on the effects of annealing temperature and Fe-doping concentration on the crystallinity, microstructure, ferroelectric and dielectric properties of PMN-PT thin films. 2% Fe-doped PMN-PT thin film annealed at 650 °C exhibits high (111) preferred orientation, high remanent polarization (*P_r_* = 23.1 μC/cm^2^) and low coercive voltage (Ec = 100 kV/cm). BiFeO_3_ thin films were doped with Sm by Wang et al. [3]. They discussed the changes of electrical characteristics in terms of phase transition and oxygen vacancies.

Van der Waals (vdW) layered ferroelectric materials have become a promising research branch in condensed matter physics. As the main factor affecting the ferroelectric property is the strong depolarization in ultrathin ferroelectric film, herein, Jia et al. [4] investigated the ferroelectric and piezoelectric properties at different thicknesses. Furthermore, the constructed Pt/CuInP_2_S_6_/Au ferroelectric tunnel junction with a 2 nm-thick functional layer has superior continuous current modulation and self-rectification functions.

In recent years, ferroelectric energy storage thin film is emerging as a key enabler for advanced pulsed power systems. However, such applications are hindered by the low energy storage density and small efficiency, so some strategies need to be explored to further improve its energy storage performance. Wang et al. [5] design a multilayer structure of Ba_0.5_Sr_0.5_TiO_3_/0.4BiFeO_3_-0.6SrTiO_3_ on flexible mica substrate. A good balance between energy density and efficiency can be reached by utilizing the interface engineering. In addition, the authors describe a cost-effective and facile fabrication of flexible ferroelectric thin films. The energy storage performance of bismuth layer-structured ferroelectric (BLSF) compounds of CaBi_4_Ti_4_O_15_ modified by Bi(Fe_0.93_Mn_0.05_Ti_0.02_)O_3_ was studied by Liu et al. [6]. In their work, Bi(Fe_0.93_Mn_0.05_Ti_0.02_)O_3_ played a very important role in enhancing the polarization and optimizing the shape of ferroelectric hysteresis loop.

Magnetic metamaterials are deemed to be a promising candidate as a high-quality information carrier. Since the graded magnonic refractive index can be created by modification of the material properties, Zhuo et al. [7] theoretically propose a ferromagnet/heavy metal bilayer magnonic metamaterial, and modulate the refractive index of spin waves with the inhomogeneous Dzyaloshinskii–Moriya interaction (DMI). The authors further study spin-wave refraction and reflection at the interface between two magnetic media with different DMI and build a generalized Snell’s law of spin waves.

Last but not least, three papers of this Special Issue address the applications involving piezoelectric photocatalyst, humidity sensor, and metal oxide semiconductor. Piezoelectric catalysis is an efficient and environmentally friendly dye degradation method. Li et al. [8] synthesized the BiOCl/NaNbO_3_ piezoelectric composites, and investigated the mechanism of piezocatalysis, photocatalysis, and their synergy effect of BN-3 composite. This work provides a new strategy to improve the environmental pollutant remediation efficiency of piezoelectric composites. Perovskites with the formula of AMX_3_ have a potential use for probes for sensing of humidity due to the heavy dependence of electrical characteristics on environmental moisture. Therefore, Zhao et al. [9] carried out relevant research on the humidity sensitivity of CH_3_NH_3_PbI_3_ perovskite. The CH_3_NH_3_PbI_3_-based humidity sensor shows the excellent humidity sensitive properties, and the authors ascribed it to water-molecule-induced enhancement of the conductive carrier concentration. As the COMS feature sizes continue to decrease, it is urgent to use a high-k material to replace the current SiO_2_ gate dielectrics. Lu et al. [10] systematically investigated the effect of atomic layer deposition-derived laminated interlayer on the interface chemistry and transport characteristics of sputtering-deposited Sm_2_O_3_/InP gate stacks. They demonstrated that Sm_2_O_3_/Al_2_O_3_/InP stacked gate dielectric is a promising candidate for InP-based metal oxide semiconductor field-effect-transistor devices in the future.

In this Special Issue, the range of themes addressed is certainly not exhaustive. With the scope of application of nanostuctured ferroic materials broadening rapidly, further developments on the aspects of theoretical simulation, synthetic technique, advanced characterization, and multi-functional combination are expected.
